# Determinants of Impaired Health-Related Quality of Life in Older Italian Outpatients with Type 2 Diabetes

**DOI:** 10.3390/jcm14176118

**Published:** 2025-08-29

**Authors:** Adrianapia Maria Lamedica, Giulia Montin, Olga Protic, Luca Antognoli, Elena Tortato, Federica Turchi, Maria Paola Luconi, Alessio Menditto, Fabiola Olivieri, Liana Spazzafumo, Anna Rita Bonfigli

**Affiliations:** 1 Scientific Direction, IRCCS INRCA, 60124 Ancona, Italy; a.lamedica@inrca.it (A.M.L.); g.montin@inrca.it (G.M.); o.protic@inrca.it (O.P.); l.spazzafumo@inrca.it (L.S.); a.bonfigli@inrca.it (A.R.B.); 2 Diabetology Unit, IRCCS INRCA, 60124 Ancona, Italy; e.tortato@inrca.it (E.T.); f.turchi@inrca.it (F.T.); m.luconi@inrca.it (M.P.L.); 3 Cardiology Unit, IRCCS INRCA, 60124 Ancona, Italy; a.menditto@inrca.it; 4 Advanced Technology Center for Aging Research, IRCCS INRCA, 60124 Ancona, Italy; f.olivieri@univpm.it; 5 Department of Clinical and Molecular Sciences, Università Politecnica delle Marche, 60121 Ancona, Italy

**Keywords:** type 2 diabetes, outpatients, older adults, Health-Related Quality of Life, EQ-5D-3L, EQ-5D index, comprehensive geriatric assessment

## Abstract

**Introduction**: Health-Related Quality of Life (HRQOL) is a key indicator of how chronic conditions such as type 2 diabetes (T2D) affect an individual’s overall well-being. This study explored the relationship between HRQOL and health-related data in older T2D participants. **Methods**: This retrospective study analyzed data from 987 participants with T2D who underwent checkups at the IRCCS INRCA Hospital, Ancona, Italy. Participants with an EQ-5D-3L index score of 1 were classified as having “no problems” in any dimension, and those with a value less than 1 were categorized as having “any problems”. **Results**: The mean age was 76.5 years (SD ± 4.5), and 46.5% were male. The “any problems” group (n = 795) was older and had longer diabetes duration, lower educational attainment, higher total cholesterol, higher triglycerides, and a higher Body Mass Index (BMI) with respect to the “no problems” group (n = 195). Multivariate logistic regression identified female sex (OR 2.14, 95% CI: 1.24–3.70), higher BMI (OR 1.07, 95% CI: 1.01–1.14), and depressive symptoms by the 5-item mini-Geriatric Depression Scale (mini GDS 5) (OR 2.43, 95% CI: 1.84–3.21) as significantly associated with lower HRQOL. Conversely, higher scores of the Instrumental Activities of Daily Living (IADL) (OR 0.77, 95% CI: 0.65–0.91) were related to better HRQOL. **Conclusions**: Female sex, overweight status, depressive symptoms, and lower physical performance are associated with impaired HRQOL in older Italian T2D outpatients. In particular, the EQ-5D-3L index provides a comprehensive index associated with depressive symptoms and low mobility.

## 1. Introduction

Diabetes is a prevalent condition among older adults, affecting approximately 20% of this population. It has been identified as the third leading cause of disability-adjusted life-years (DALYs) in adults aged 50 to 75 and the second leading cause in those over 75 [1]. Type 2 diabetes (T2D) can negatively impact activities of daily living, self-care practices, the risk of developing long-term complications, overall well-being, and healthcare expenditures [2]. This chronic disease can cause long-term damage and failure of various organs, particularly the eyes, kidneys, nerves, heart and blood vessels with a negative impact on life expectancy and quality of life [3]. Prior research has identified several factors influencing Health-Related Quality of Life (HRQOL), including physical aspects such as diabetes complications and comorbidities [4]; mental health components like diabetes-related distress and depression and psychosocial elements such as social support and coping strategies [5,6]. Over the past few decades, assessing HRQOL has become essential in clinical research, public health planning and healthcare services [7]. HRQOL is a fundamental indicator for assessing how the management of chronic diseases such as diabetes affects an individual’s well-being [8]. Questionnaires are a valuable tool for assessing quality of life with the European Quality of Life 5 Dimensions 3 Level Version (EQ-5D-3L) being one of the most widely used instruments in the literature [9,10]. The EQ-5D-3L consists of 2 parts: the EQ-5D descriptive system and the EQ visual analog scale (EQ-VAS). Despite the widespread use of the EQ-VAS, the algorithm that generates the EQ-5D-3L Index a crucial summary score of perceived health status, should be prioritized in research. This index remains underutilized, despite preference weights having been validated in several countries including Italy [11]. In particular, the EQ-5D-3L Index is a fundamental tool for assessing the quality of life in older adults with T2D, offering a comprehensive, standardized and useful measure for both clinical practice and clinical research [12]. It creates a single, standardized score from EQ-5D-3L responses. This enables simple comparison of health status across diverse patient groups. Given the multiple complications and comorbidities that impact the quality of life in older adults with T2D, the EQ-5D-3L index provides a more comprehensive measure than single dimensions, as it accounts for the relative importance of each dimension to the patient.

Despite the EQ-5D-3L index offering a comprehensive and preference-weighted measure of individuals’ overall health, few studies in the literature have investigated the association between this index and clinical data in Italian outpatients with diabetes. This study aims to investigate the relationship between EQ-5D-3L index and demographic and clinico-geriatric factors among a large cohort of older Italian T2D outpatients.

## 2. Methods

### 2.1. Participants

This retrospective study analyzed data from older adults with T2D, mean age 76.5 (SD ± 4.5) years, who attended routine outpatient checkups at the Diabetology Unit of the IRCCS INRCA Hospital in Ancona, Italy, between 2014 and 2018. The study received ethical approval from the IRCCS INRCA Hospital’s Ethical Committee (reference number: CE-INRCA-18013), which granted a waiver of informed consent due to the use of deidentified, retrospective data. Glycemic control, risk factor management, and treatment were determined by diabetologists in accordance with standard clinical practice guidelines consistent with the joint Associazione Medici Diabetologi (AMD) and Società Italiana di Diabetologia (SID) Italian guidelines for the treatment of diabetes mellitus [13]. Comprehensive geriatric assessments were routinely performed to personalize treatment for older participants, as recommended by AMD-SID [14]. Participants with type 1 diabetes, secondary diabetes, acute illness, or severe intercurrent illnesses were excluded. Of the 1006 participants with diabetes enrolled, 19 participants with severe intercurrent disease were excluded.

### 2.2. Variables

#### 2.2.1. General Characteristics

The following variables were collected from participants enrolled in this study: ociodemographic data, anthropometric data, medical history, clinical laboratory parameters.

#### 2.2.2. T2D Features

To characterize the clinical profile of diabetes, we collected a series of specific data for each participant: T2D duration, glycated hemoglobin (HbA1c), fasting glycemia, microalbuminuria, diabetic complications, antidiabetic drugs.

#### 2.2.3. HRQOL

HRQOL was measured by EQ-5D-3L. The 3-Level Version of the EQ-5D (EQ-5D-3L) was introduced in 1990 by the EuroQol Group. The EQ-5D-3L Essentially Consists of 2 Parts: The EQ-5D Descriptive System and the EQ Visual Analog Scale (EQ-VAS) [15].

EQ-5D descriptive system: this section comprises five dimensions: Mobility, Self-care, Daily Activities, Pain/Discomfort and Anxiety/Depression. Each dimension has three levels of severity: “no problems”, “mild problems” and “severe problems”. The EQ-5D descriptive system provides a health status profile that can be converted into a single index value using country-specific value sets. In this study. the EQ-5D-3L index was calculated using preference weights specific to the Italian population. The scoring function is derived from econometric models, whose coefficients are used for utility estimation. In utility calculation, the coefficients obtained from the models were transformed by subtracting them from 1. The five dimensions of the EQ-5D-3L (Mobility, Self-care, Daily Activities, Pain/Discomfort, Anxiety/Depression) were transformed into dummy variables: each dimension was assigned a value of 1 if the reported severity level is 2 or 3 (depending on the variable considered), and 0 otherwise. For better alignment with the preferences expressed by the general population, supplementary variables were also included in the calculation. In particular, the Italian formula includes the use of the variable “number of dimensions at level 2 or 3 beyond the first” (D1) [11].

EuroQol Visual Analogue Scale (EQ-VAS): this part is a vertical scale from 0 to 100, where 0 represents ‘worst imaginable health’ and 100 represents ‘best imaginable health’. Participants indicate their general health status on the EQ-VAS scale.

#### 2.2.4. Geriatric Assessment

The Mini-Geriatric Depression Scale (Mini-GDS 5) is a five-item screening tool used to identify signs suggestive of depression. A total score of 0–1 indicates a normal result, while a score of 2 or higher suggests the presence of depression [16].

The Mini Nutritional Assessment (MNA) is a nutritional status assessment tool that consists of two phases: a screening phase and a global assessment phase. Based on the total score obtained (from the sum of screening and global assessment scores), participants are classified into three categories: Malnourished: score less than 17; At the risk of malnutrition: score between 17 and 23.5; Well-nourished: score between 24 and 30. The standard procedure is that the global assessment phase of the MNA is performed only in participants who do not exceed the screening threshold [17].

The Basic Activities of Daily Living (ADL) assess an individual’s capacity to perform essential self-care tasks, such as bathing, dressing, toileting, and eating. The final score ranges from 0 to 6, where 0 indicates total dependence and 6 represents complete autonomy [18].

The Instrumental Activities of Daily Living (IADL) assess an individual’s ability to perform complex tasks necessary for independent living, such as shopping, cooking, managing finances and using transportation. The final score ranging from 0 to 8 indicates the level of autonomy: 0 represents total dependence, while 8 corresponds to complete autonomy [19].

The Short Portable Mental State Questionnaire (SPMSQ), also known as the Pfeiffer Test, is a widely utilized, rapid, and straightforward screening instrument comprising 10 items designed to evaluate the potential presence of cognitive impairment, particularly within the geriatric population. This assessment tool probes various cognitive domains, including the following: temporal and spatial orientation: assessed thought inquiries pertaining to the current date, day of the week, and geographical location; long-term memory: evaluated via questions concerning personal information such as telephone number, address, and mother’s maiden name; concentration and calculation: investigated through a serial substraction task.

The SPMSQ serves as an effective tool for the preliminary identification of individuals warranting subsequent comprehensive diagnostic evaluation. While the test results are fundamentally interpreted based on the number of correct responses, a thorough understanding of the scoring methodology is crucial, as a lower score (i.e., fewer correct answers) is indicative of a higher probability of cognitive impairment. The scoring of the SPMSQ is determined by assigning one point for each incorrect response across the ten questionnaire items. The raw score thus represents the total number of errors committed, with a potential range from 0 to 10. Subsequently, this raw score undergoes correction based on the subject’s educational attainment. Specifically, for individuals with eight years of formal education or less, one point is added to the raw score. Conversely, if the educational duration exceeds eight years, one point is subtracted from the raw score. For those with precisely eight years of education, no correction is applied. The final adjusted score is then interpreted: a higher number of errors in this corrected score signifies a more pronounced degree of cognitive impairment [20].

Short Physical Performance Battery (SPPB) is a geriatric tool that assesses the physical functionality of the lower limbs in older individuals through three tests: balance in various positions, walking speed over 4 m and the ability to rise from a chair 5 times. The score ranges from 0 to 12, where a higher score indicates better functionality. The use of the SPPB scale enables the identification of risk of decline and disability, monitoring changes over time and evaluating the effectiveness of rehabilitation interventions. In practice, it is a quick and reliable way to understand how well older adults can move [21].

### 2.3. Statistical Analysis

Descriptive statistics were expressed as percentages for categorical variables, mean and standard deviation (±SD) for continuous variables. Participants with an EQ-5D-3L index value of 1 were classified as having “no problems” on any dimension (“no problems” group), while participants with an EQ-5D-3L index value lower than 1 were classified as having “any problems” on at least one of the five dimensions (“any problems” group). A univariate analysis was conducted to evaluate the association of each independent variable with the dependent variable, EQ-5D-3L index groups. Continuous variables were assessed using the independent samples *t*-test, while categorical variables were analyzed using chi-squared test. All variables that demonstrated statistical significance in this preliminary analysis were included in the multivariable logistic regression model.

The independent variables included in the model were: age, sex (categorical: male/female, with male as the reference category), education, T2D duration, Body Mass Index (BMI), Total Cholesterol, Triglycerides, IADL score, SPMSQ score, MNA score, GDS score, and SPPB score.

All selected independent variables were entered simultaneously into the logistic regression model. Odds ratios (ORs) with their 95% confidence intervals (CIs) and associated *p*-values, are reported. A *p*-value of <0.05 was considered statistically significant. Statistical analyses were performed using IBM SPSS Statistics version 24 for Windows.

## 3. Results

This study involved 987 outpatients with T2D, with a mean age of 76.5 (SD ± 4.5) years, and a male prevalence of 46.5%. The sociodemographic and clinical characteristics are reported in Table 1. The mean of T2D duration was 16.4 years (SD ± 11.1) and the mean HbA1c value was 7.4 (SD ± 1.2). A total of 195 participants (19.8%) achieved the maximum EQ-5D-3L index score of 1, indicating no reported problems (“no problems” group). The remaining 795 participants (80.2%) reported at least one problem on the EQ-5D-3L (“any problems” group), with a mean index score of 0.80 ± 0.15. This group was predominantly female (59.7%). The EQ visual analog scale (EQ-VAS) score was significantly lower in the “any problems” group compared to the “no problems” group (Mean ± SD values were 62.6% ± 18.5 vs. 76.1% ± 13.3, respectively, *p* < 0.001).

As shown in Table 1, participants with EQ-5D-3L problems (“any problems” group) exhibited significantly older age, longer T2D duration, lower educational years, a higher percentage of females and a higher average BMI compared to the participants in the “no problems” group. Furthermore, respondents reporting EQ-5D-3L problems were found to have elevated mean levels of Total Cholesterol (175.7 ± 41.2 mg/dL vs. 166.4 ± 35.5 mg/dL, *p* = 0.010), Triglycerides (134.7 ± 73.3 mg/dL vs. 118.5 ± 59.6 mg/dL, *p* = 0.011), and BMI (28.9 ± 4.9 vs. 27.7 ± 3.8, *p* < 0.001) relative to those without EQ-5D-3L problems.

**Figure 1:**
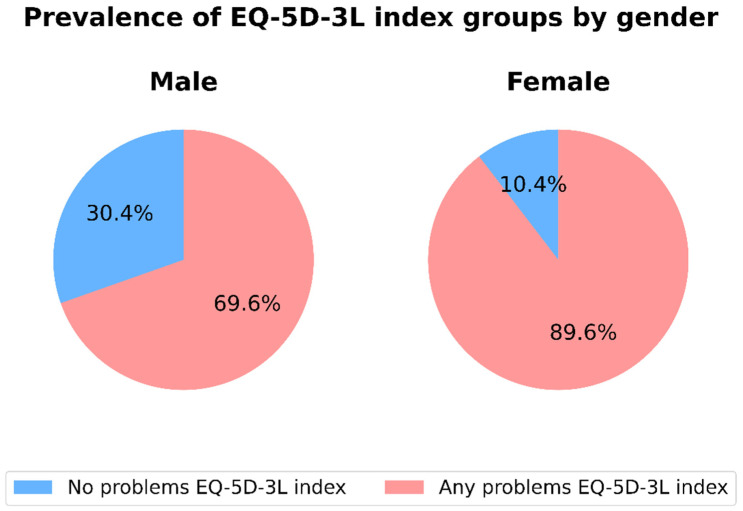
Distribution of self-reported problems using EQ-5D-3L index in male and female respondents with T2D. (Chi-squared test = 62.635; *p* < 0.001).

As shown in Figure 1, the proportion of respondents reporting any problems on the EQ-5D-3L index was significantly different between males and females compared to those reporting no problems (Chi-squared test = 62.635, *p* < 0.001).

**Figure 2:**
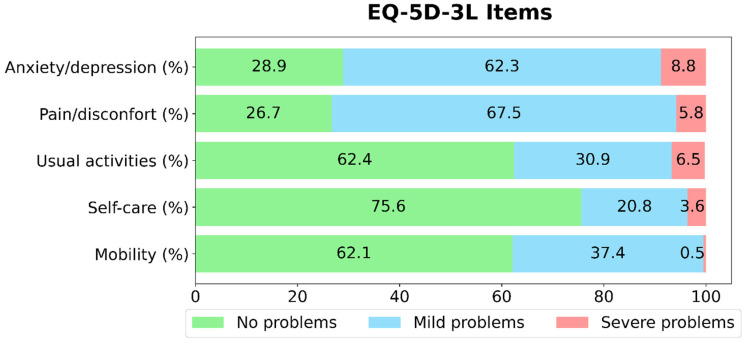
Percentage distribution into “no problems”, “mild problems”, and “severe problems” levels across the five dimensions of the EQ-5D-3L questionnaire in T2D respondents.

The percentage distribution of patient responses across the five dimensions of the EQ-5D-3L questionnaire: Anxiety/Depression, Pain/Discomfort, Daily Activities, Self-care, and Mobility. For each dimension, the data are stratified into three categories: “no problems”, “mild problems” and “severe problems” (Figure 2).

No problems were reported by 62.1% of participants regarding Mobility, 75.6% regarding self-care, 62.4% regarding Daily Activities, 26.7% regarding Pain and Discomfort and 28.9% regarding Anxiety and Depression.

The results of the comprehensive geriatric assessment (CGA) are reported in Table 2. The comparison of CGA outcomes between individuals with diabetes of the “no problems” group and “any problems” group reveals statistically significant differences across several key domains. Specifically, the “any problems” group exhibited poorer functional status (IADL), cognitive function (SPMSQ), nutritional status (MNA), and physical performance (SPPB) compared to those reporting no problems (all *p* < 0.001). Furthermore, the “any problems” group reported significantly higher depressive symptoms, as indicated by the GDS (*p* < 0.001). No significant difference was observed between the two groups in ADL scores (*p* = 0.836).

In the multivariable logistic regression analysis (Table 3), we found no significant difference between the two groups for age, years of education, T2D duration, total cholesterol, triglycerides, SPMSQ and MNA. Female gender, higher BMI scores, lower IADL scores (indicating greater functional impairment), higher GDS scores (reflecting more severe depressive symptoms), and lower SPPB scores (suggesting poorer physical performance) were all identified as significant independent factors of belonging to the having “any problems” group (OR [95% CI]: Female gender: 2.14 [1.24–3.70], *p* = 0.006; BMI score: 1.07 [1.01–1.14], *p* = 0.016; IADL score: 0.77 [0.65–0.91], *p* = 0.002; GDS score: 2.43 [1.84–3.21], *p* < 0.001; SPPB score: 0.73 [0.65–0.83], *p* < 0.001).

## 4. Discussion

The present study aimed to explore the association between HRQOL and demographic and clinico-geriatric factors in a large cohort of older Italian outpatients with T2D.

We chose the EQ-5D-3L index for HRQOL analysis because it offers significant advantages over individual items or the EQ-VAS scale [22]. It condenses five health dimensions into a single, comprehensive, and interpretable numerical value, reflecting overall perceived health. Crucially, the index incorporates population-based value sets, which are essential for economic evaluations and for capturing the broader societal impact of health states. This integrated approach is more sensitive to subtle health changes and simplifies analysis, providing a robust, preference-weighted measure of HRQOL [23,24].

The results of our analysis showed that female sex, overweight status, the presence of depressive symptoms, and reduced physical performance were associated with poorer quality of life. Specifically, women reported a significantly higher prevalence of problems compared to men, indicating a lower perceived health-related quality of life among female participants, consistent with the previous literature [25].

The literature confirms this difference, which may stem from a complex interplay of biological factors, higher rates of comorbid conditions, and specific psychosocial stressors or higher health expectations reported by older women [23]. Recognizing these gender-specific vulnerabilities is critical for designing targeted interventions.

Furthermore, in our study, we observed a strong association between overweight status and poorer HRQOL, aligning with well-established findings in various populations [26]. The distribution of responses across the five EQ-5D-3L dimensions revealed that participants frequently reported problems related to pain/discomfort and anxiety/depression, even when self-care, daily activities, and mobility were relatively preserved. This highlights how the subjective and often less visible dimensions of health profoundly impact well-being in this cohort of outpatients.

The strong relationship between depressive symptoms and reduced HRQOL in older adults with T2D is a consistent finding in our study, corroborating previous research [27,28]. Depression, often underdiagnosed in this population, significantly exacerbates the burden of diabetes, impacting self-management, treatment adherence, and overall quality of life [29]. T2D is a known contributor to physical disability in older adults [30,31,32], and our data underscore how mobility limitations and reduced functional independence are associated with diminished quality of life, potentially leading to social isolation and emotional distress [33]. These findings align with recent research highlighting the relationship among physical function, mental health, and HRQOL in geriatric populations [34].

The identification of factors associated with compromised HRQOL carries crucial practical implications for the clinical management and public health strategies targeting older outpatients with T2D for the following reasons: firstly, our results suggest that routine screening for perceived quality of life using the EQ-5D-3L should be prioritized in diabetology units for older outpatients with diabetes. These data could aid in stratifying patients based on impaired HRQOL, thereby allowing for more personalized preventative strategies.

This is important to identify low HRQOL, which in this specific population target, is primarily associated with depressive symptoms. Early detection can facilitate timely referral to mental health professionals and the initiation of appropriate interventions (such as psychotherapy or pharmacotherapy), which are crucial for maintaining both mental well-being and HRQOL [35]. Secondly, our study also found that compromised HRQOL is associated with reduced physical performance. The literature describes various interventions aimed at improving physical performance, including promoting tailored exercise programs that have a positive impact on healthcare system costs [36] and encourage a shift toward active lifestyles in this population [37]. Multimodal intervention including exercise programs can improve perceived HRQOL [38]. The ultimate goal is to provide clinicians with the tools to more effectively tailor treatment plans and maintain the overall well-being of their patients.

The EQ-5D-3L is a brief, quick, and easy self-administered test, making it highly suitable for an outpatient setting. The test provides valuable information on depressive and functional status, which is beneficial for the clinical management of older participants with T2D [39].

Patient management represents a personalized and team-based approach. Therapeutic decisions, guided by scientific evidence, must be timely and consider the patient’s preferences, comorbidities, and personal circumstances. This process combines lifestyle modifications, such as diet and physical exercise, with pharmacological therapy when necessary. The ultimate goal is to continuously improve the quality of care through constant data evaluation, thereby ensuring a better quality and duration of life for respondents [40].

This study has several limitations that warrant consideration. Firstly, its retrospective design precludes the establishment of causal relationships, allowing only for the identification of associations. Longitudinal studies are needed to explore how these factors influence the temporal dynamics of changes in HRQOL. Secondly, the study population comprises outpatients attending a single IRCCS INRCA Hospital in Ancona, Italy. While this provides valuable insights into a specific clinical setting, it may limit the representativeness of the sample to the broader population of older Italian adults with T2D, particularly those who do not regularly access specialized diabetology care or reside in different geographical/socioeconomic contexts. Therefore, the generalizability of our results to community-dwelling older adults with T2D outside of this specialized outpatient setting, or to different healthcare systems, should be interpreted with caution. Future studies involving more diverse and larger cohorts are needed to confirm these findings.

## 5. Conclusions

In conclusion, female sex, overweight status, depressive symptoms, and lower physical performance are associated with impaired HRQOL among older Italian outpatients with T2D. The EQ-5D-3L index represents a comprehensive, preference-weighted, and interpretable measure of HRQOL in this population. The EQ-5D-3L index captures the multidimensional nature of health status and its association with key geriatric vulnerabilities, such as depression and impaired mobility.

In the context of lifestyle modifications, our findings underscore the importance of a multifaceted approach that goes beyond the traditional prescription of diet and exercise. Clinical strategies should be enriched with targeted educational and support programs. These could include structured therapeutic education sessions conducted by a multidisciplinary team (e.g., diabetologists, dietitians, and physical therapists) to provide personalized guidance [41]. The use of focus groups could also facilitate peer support and shared learning [42]. The dissemination of educational materials, such as flyers or digital content, is another valuable tool for reinforcing key messages about the importance of physical activity and healthy eating [43]. By implementing these patient-centered strategies into routine clinical care, we can move beyond generic advice to achieve a more significant and sustainable improvement in patient well-being, particularly for those with reduced quality of life due to compromised physical performance or depressive symptoms.

## Figures and Tables

**Table 1:** Participant characteristics and comparison of sociodemographic and clinical features between “no problems” and “any problems” EQ-5D-3L index groups.

	“No Problems” Group * (n = 195)	“Any Problems” Group ** (n = 795)	All Subjects (n = 987)	*p* Value
Age, years	75.3 (4.0)	76.8 (4.6)	76.5 (4.5)	<0.001
Sex, female, n (%)	55 (28.2)	475 (59.7)	530 (53.7)	<0.001
Education, years	7.9 (3.8)	6.8 (3.6)	6.9 (3.7)	<0.001
T2D duration, years	14.5 (10.2)	16.8 (11.2)	16.4 (11.1)	0.008
BMI, kg/m^2^	27.7 (3.8)	28.9 (4.9)	28.7 (4.7)	<0.001
Diastolic Blood Pressure, mmHg	76.6 (9.5)	76.8 (9.6)	76.8 (9.6)	0.781
Systolic Blood Pressure, mmHg	139.2 (18.7)	142.2 (18.4)	141.7 (18.4)	0.095
Fasting glucose, mg/dL	143.9 (37.1)	145.8 (42.3)	145.4 (41.4)	0.586
HbA1c, %	7.4 (1.2)	7.5 (1.2)	7.5 (1.2)	0.432
Microalbuminuria, mg/L	45.4 (111.4)	38.9 (105.0)	40.3 (106.4)	0.453
Creatinine, mg/dL	1.03 (0.35)	1.07 (0.63)	1.06 (0.59)	0.448
Total Cholesterol, mg/dL	166.4 (36.5)	175.7 (41.2)	173.8 (40.5)	0.010
HDL Cholesterol, mg/dL	50.0 (13.9)	50.8 (13.9)	50.6 (13.9)	0.517
Triglycerides, mg/dL	118.5 (59.6)	134.7 (73.3)	131.3 (70.9)	0.011
ALT (IU/L)	21.9 (10.3)	21.5 (12.5)	21.6 (12.2)	0.698
AST (IU/L)	20.5 (10.3)	20.2 (12.4)	20.3 (12.1)	0.794
Uric Acid, mg/dL	5.3 (1.4)	5.4 (2.0)	5.4 (1.9)	0.615
EQ-VAS score	76.1(13.3)	62.6 (18.5)	65.2(18.5)	<0.001

* “No problems” Group: participants with EQ-5D-3L Index = 1; ** “Any problems” Group: participants with EQ-5D-3L Index < 1; T2D = Type 2 Diabetes; BMI = Body Mass Index; HbA1c = glycosylated hemoglobin; HDL = high-density lipoprotein; ALT = alanine aminotransferase; AST = aspartate amino-transferase; EQ-VAS = EuroQol Visual Analogue Scale. Data were expressed as mean ± SD, or n (%).

**Table 2:** Comprehensive geriatric assessment of T2D respondents stratified according to EQ-5D-3L index groups.

	“No Problems” Group *	“Any Problems” Group **	All Subjects	*p* Value
ADL	5.63 (0.80)	5.61 (0.88)	5.62 (0.86)	0.836
IADL	6.71 (1.54)	5.98 (2.13)	6.12 (2.05)	<0.001
SPMSQ	9.50 (0.76)	8.94 (1.60)	9.05 (1.49)	<0.001
GDS	0.43 (0.74)	1.51 (1.45)	1.30 (1.41)	<0.001
MNA	27.25 (2.06)	25.77 (2.77)	26.05 (2.71)	<0.001
SPPB	9.59 (1.70)	6.65 (3.43)	7.22 (3.37)	<0.001

* “No problems” Group: participants with EQ-5D-3L Index = 1; ** “Any problems” Group: subjects with EQ-5D-3L Index < 1; ADL = Basic Activities of Daily Living; IADL = Instrumental Activities of Daily Living; SPMSQ = Short Portable Mental Status Questionnaire; GDS = Geriatric Depression Scale; MNA = Mini Nutritional Assessment; SPPB = Short Physical Performance Battery. Data were expressed as mean ± SD, or n (%).

**Table 3:** Multiple logistic regression analysis: factors associated with impaired HRQOL (EQ-5D-3L Index < 1).

	OR	95%LL	95%UL	*p* Value
Age, years	1.01	0.96	1.07	0.786
Sex, female	2.14	1.24	3.70	0.006
Education, years	0.99	0.94	1.05	0.869
T2D duration, years	1.01	0.98	1.03	0.473
BMI, kg/m^2^	1.07	1.01	1.14	0.016
Total Cholesterol, mg/dL	1.01	0.99	1.01	0.180
Triglycerides, mg/dL	1.00	0.99	1.00	0.867
IADL	0.77	0.65	0.91	0.002
SPMSQ	0.88	0.68	1.13	0.346
GDS	2.43	1.84	3.21	<0.001
MNA	0.93	0.83	1.05	0.298
SPPB	0.73	0.65	0.83	<0.001

OR = odds ratio; BMI = Body Mass Index; T2D = Type 2 Diabetes; IADL = Instrumental Activities of Daily Living; SPMSQ = Short Portable Mental Status Questionnaire; MNA = Mini Nutritional Assessment; GDS = Geriatric Depression Scale; SPPB = Short Physical Performance Battery.

## Data Availability

The datasets generated and/or analyzed during the current study are not publicly available due to privacy and ethical restrictions but are available from the corresponding author on reasonable request.
